# Blood Flow Restriction Enhances Rehabilitation and Return to Sport: The Paradox of Proximal Performance

**DOI:** 10.1016/j.asmr.2021.09.024

**Published:** 2022-01-28

**Authors:** Corbin Hedt, Patrick C. McCulloch, Joshua D. Harris, Bradley S. Lambert

**Affiliations:** Houston Methodist Orthopedics & Sports Medicine, Houston, Texas, U.S.A.

## Abstract

The use of blood flow restriction (BFR) within rehabilitation is rapidly increasing as further research is performed elucidating purported benefits such as improved muscular strength and size, neuromuscular control, decreased pain, and increased bone mineral density. Interestingly, these benefits are not isolated to structures distal to the occlusive stimulus. Proximal gains are of high interest to rehabilitation professionals, especially those working with patients who are limited due to pain or postsurgical precautions. The review to follow will focus on current evidence and ongoing hypotheses regarding physiologic responses to BFR, current clinical applications, proximal responses to BFR training, potential practical applications for rehabilitation and injury prevention, and directions for future research. Interestingly, benefits have been found in musculature proximal to the occlusive stimulus, which may lend promise to a greater variety of patient populations and conditions. Furthermore, an increasing demand for BFR use in the sports world warrants further research for performance research and recovery.

**Level of Evidence:**

Level V, expert opinion.

Blood flow restriction (BFR) training has received increasing attention throughout scientific literature and mainstream media as its significance grows in the rehabilitation industry. Reported benefits include improved muscle strength,^1^, ^2^, ^3^, ^4^ mitigation of post-traumatic atrophy,^2^^,^^5^^,^^6^ heightened neuromuscular activity,^7^ decreased pain signaling,^8^, ^9^, ^10^ and increased bone mineral density.^11^ Importantly, these effects have been most reported to occur in the regions distal to the site of occlusion (commonly applied at the most proximal points of the extremities). However, recent studies have highlighted promise in benefit to the proximal structures as well.^12^, ^13^, ^14^, ^15^

When considering the shoulder or hip, one can appreciate the delicate balance in mobility and stability required by the proximal limbs. The glenohumeral and scapulothoracic regions provide a complex synergy of muscle actions in an effort to push, pull, and rotate to an exceedingly high degree, especially with high-level sports such as throwing, gymnastics, and weight-lifting.^16^ The lumbopelvic region ensures a similar means of dynamic stability, but typically in more weight-bearing positions and with impact-related activities such as yoga, running, jumping, or dancing.^17^ In the rehabilitation and physical therapy environments, the proximal aspect of a given limb assumes a major role in most rehabilitation programs. While the use of BFR limits occlusion to distal limbs beyond a tourniquet cuff, the potential for proximal benefit seems promising. However, further research is needed to characterize the exact nature of proximal responses of BFR training and to what degree it may be applied in clinical rehabilitation settings or for injury prevention.

Originally adapted from early studies on muscular occlusion, the initial literature heavily focused on the effectual consequences of vascular manipulation with exercise.^18^, ^19^, ^20^ The moniker KAATSU training is prominent from research in the late 1990s and early 2000s. These works set the foundation for modern practices and clinical paradigms. While the technology was rudimentary and the occlusive stimuli were rather ambiguous, these studies have allowed for the refinement of current protocols to ensure safety and efficacy with clinical practice.

As rehabilitation professionals adopt and implement BFR into their practice, we must be fastidious in our evaluation of the science. Often with novel technology and techniques, clinical adoption can be antecedent to strong evidence and systematic study. Although BFR is commonly applied in clinical settings, a high degree of speculation remains present in the literature regarding the efficacy of BFR for tissues proximal to the site of occlusion and the potential mechanisms that may play a role. The review to follow will focus on current evidence and ongoing hypotheses regarding physiologic responses to BFR, current clinical applications, proximal responses to BFR training, potential practical applications for rehabilitation and injury prevention, and directions for future research.

## Physiologic Mechanisms: An Overview

In summary, the combination of BFR and low-intensity resistance exercise (BFR-LIX; often defined as exercise performed below ∼30% maximal effort) has often been reported to elicit physiologic responses that are somewhat similar to high-intensity exercise (>∼70% maximal effort) with regards to exercise-induced muscle anabolism and various improvements in muscle performance (e.g., functional capacity, strength).^2^^,^^21^, ^22^, ^23^ The clinical interest in this phenomenon relates to the capacity to provide a sufficient exercise stimulus with reduced mechanical load that may be performed in the early stages following injury or in the early postoperative period after surgery.^1^^,^^11^^,^^24^^,^^25^

Although the need for further research remains, the acute and chronic skeletal muscle responses to BFR have heavily characterized in numerous basic and applied investigations as well as summarized in a number of systematic reviews and meta-analyses with regard to musculature distal to the site of occlusion.^2^^,^^21^^,^^26^, ^27^, ^28^, ^29^, ^30^, ^31^, ^32^, ^33^ To summarize, the skeletal muscle response to BFR-LIX has been hypothesized to be caused by a combination of intramuscular metabolic stress sensing, metabolite accumulation, mechano-transduction signaling (via muscle contractile activity and cell swelling caused by occlusion), hormonal responses to exercise, intracellular hypoxia, and inflammatory signaling mechanisms.^21^^,^^31^^,^^34^^,^^35^

## Effects of BFR Distal to the Occlusion Site

### Skeletal Muscle Responses to Resistance Exercise

Regarding the responses of skeletal muscle to BFR exercise and training, the majority of investigations have focused on musculature distal to the site of occlusion. To summarize, BFR-LIX has been shown to acutely induce skeletal muscle anabolism through stimulation of mammalian target of rapamycin complex 1 and mitogen-activated protein kinase intracellular signaling pathways, which are both key regulators of skeletal muscle growth and/or remodeling.^29^, ^30^, ^31^^,^^36^^,^^37^ Both pathways also are required to elicit maximal protein synthesis rates and can operate in an independent or integrated fashion responding to contractile activity (exercise), cell stress, nutrition, intracellular energy availability, hormone signaling (e.g., insulin and insulin-like growth factors, [IGFs]), and cytokine/myokine signaling. In the case of injury or the postoperative period, anabolic signaling has been observed to be suppressed due to unloading/inactivity resulting in a loss of muscle mass and function.^28^^,^^32^^,^^38^, ^39^, ^40^, ^41^ Increased protein degradation also has been implicated to play a role in the sarcopenic effects of unloading.^42^ Therefore, because of its anabolic effects with reduced loading, BFR-LIX has been hypothesized to be a suitable candidate for combating postoperative sarcopenia and loss of function. As a result of occlusion, BFR-LIX elicits acute intramuscular hypoxia and an accumulation of lactate (local and systemic) with corresponding decreases in pH and increased in CO_2_.^43^, ^44^, ^45^ These responses have been observed to facilitate postexercise increases in anabolic signaling (centrally and locally) as well as significantly influence metabolic adaptations to exercise training.^46^, ^47^, ^48^

In addition to the production of metabolites such as lactate and CO_2_ into circulation during strenuous exercise, skeletal muscle has also been observed to release a host of myokines that have the capacity to act in an autocrine, paracrine, and endocrine manner.^49^, ^50^, ^51^ Cumulatively, it is hypothesized that immediately following the occlusive stimulus, the release of these effectors into circulation may stimulate anabolism (for muscle, bone, and connective tissue) locally as well as systemically.^21^ For example, BFR-LIX has been observed to acutely increase systemic growth hormone (GH)^29^^,^^30^^,^^50^^,^^52^ release similar to high-intensity exercise in response to elevations in systemic lactate concentrations among other central mechanisms.^53^^,^^54^ Although there is ongoing debate as to the direct role that GH may have with regard to skeletal muscle responses to exercise,^31^ upregulation and release of systemic IGF-1 (a potent stimulator of mammalian target of rapamycin complex 1–mediated anabolism) as a result of GH signaling at the liver has been previously attributed to findings of both Abe et al.^55^ and Takano et al.,^56^ who observed chronic and acute increases in systemic IGF-1 following BFR-LIX training. Notably, both GH and IFGs have been shown to play an important role in satellite cell proliferation and differentiation during recovery.^57^, ^58^, ^59^ Therefore, because of the multinucleated nature of skeletal muscle, BFR may indirectly yield chronic training responses via increased potential for future growth in response to exercise.^21^^,^^31^ These systemic signaling mechanisms have been postulated to have impact in both the occluded limb undergoing BFR as well as tissues proximal to the site of occlusion (discussion to follow).^21^

In addition to local and systemic impacts on muscle, recent pilot data indicate that BFR-LIX may have a positive impact on bone and possibly connective tissue.^11^ For example, it was recently observed that during 12 weeks of rehabilitation from anterior cruciate ligament (ACL) reconstruction, the incorporation of BFR-LIX yielded significant reductions in whole limb and site-specific bone loss compared with standard rehabilitation alone.^11^ It has been hypothesized that venous occlusion may lead to fluid shifts causing increased intramedullary pressure and interstitial fluid flow within the bone.^60^ Previous observations also suggest that the response of bone to chronic exercise is potentially interlinked with skeletal muscle with regards to mechanical, systemic, and local signaling factors.^44^, ^45^, ^46^ Relatedly, myokines secreted from muscle during exercise in an intensity/stress dependent manner are known to act on bone metabolism in either pro-formation or resorption capacities.^51^^,^^61^, ^62^, ^63^, ^64^ In addition, both systemic and muscle-derived IGFs and fibroblast growth factors are also known to act directly at the muscle–bone interface.^62^^,^^65^ Lastly, BFR has been previously observed to inhibit myostatin (a negative regulator of muscle and bone anabolism) expression.^21^^,^^31^^,^^66^ Importantly, inhibition of myostatin action and/or expression has been observed to improve muscle and fracture healing following trauma.^60^^,^^66^ While further research will be required to determine cause and effect, the present data provide impactful evidence that BFR may be a suitable tool for combatting postoperative bone loss and potentially assisting with bone graft integration at the knee.

### Skeletal Muscle Activation

The overall anabolic response to resistance exercise is largely governed by the volume of work performed. This can be quantified as the total mechanical work (total sets × repetitions performed), the total metabolic cost of the exercise bout, and the amount of muscle recruited for a given exercise or set of exercises. However, when comparing BFR-LIX with standard LIX performed to fatigue, electromyographic (EMG) studies (used an indirect indicator of skeletal muscle activation) have resulted in mixed findings. For example, Wernbom et al.^67^ observed no difference in peak muscle activation (expressed as EMG amplitude; EMGa) during a lower-extremity fatigue protocol comparing BFR-LIX with LIX alone. In an upper-extremity model, Counts et al.^68^ observed no difference in EMGa when performing the same exercise under differing occlusion pressures ranging from 40% to 90% limb occlusion pressure (LOP). However, in a similar upper-extremity model evaluating fatiguing triceps extensions and biceps curls, Yasuda et al.^44^ observed increases in EMGa with increasing repetitions using the standard 30-15-15-15 protocol. Fatela et al.^69^ observed increased EMGa in the rectus femorus and vastus lateralis during leg extension exercise with increasing occlusion pressure ranging from 40% to 80% LOP. Lastly, although frequently described as being similar to high-intensity exercise, Bordessa et al.^70^ observed greater EMGa with high intensity leg extension exercise (80% 1-repetition maximum) compared with low intensity (30% 1-repetition maximum) with BFR. Using a similar exercise protocol, Teixeira et al.^71^ recently observed that during high-intensity leg extension exercise, the application of BFR reduced EMGa but did increase markers of metabolic stress, a finding similar to Dankel et al.,^72^ who observed no additive effect of BFR during high-intensity elbow flexion exercise. In summary, the degree to which BFR-LIX impacts muscle recruitment distal to the cuff requires further study. With regard to high-intensity exercise, BFR does not appear to elicit an additive effect indicating an upper threshold related to exercise intensity.

## Proximal Effects of BFR

Previous reports characterizing the local and systemic responses of BFR-LIX have increased interest in the potential proximal benefits of occlusion training. This includes responses to tissues directly proximal to the site of the occlusion, responses in the contralateral limb relative to the limb undergoing BFR-LIX, and whole-body systemic responses. Recent investigations are now beginning to provide critical insight on the efficacy of BFR for tissues located proximally to the occlusion site.

### Effects of BFR on Skeletal Muscle Directly Proximal to the Occlusion Site

Until recent years, many of the potential benefits of BFR were thought to be limited to tissues undergoing occlusion (distal to where the pressure is applied around the contracting limb). However, recent findings indicate that there may be some benefit to tissues directly proximal to the occlusion site. Although the mechanisms of action are still under a great deal of investigation, such findings have important clinical implications for the utility of BFR. In a recent study, Lambert et al.^12^ observed that following 8 weeks of low load bilateral rotator cuff training for the shoulder, BFR-LIX (performed under 50% LOP) yielded greater increases in whole limb and shoulder region muscle mass, greater improvements in muscular work capacity compared, and some greater improvements in isometric strength compared with LIX alone in healthy. untrained adults. In this study, participants performed 4 common rotator cuff strengthening exercises (cable internal rotation, cable external rotation, dumbbell scaption, and side-lying dumbbell external rotation) performing an initial set of 30 repetitions followed 2 sets of 15 repetitions and a final set performed to fatigue (trained twice per week). These findings were paired with greater EMGa observed during fatigue testing while limbs were undergoing occlusion compared to unoccluded.^12^ These results are somewhat in line with previous findings that BFR may result in greater proximal muscle activation as a result of occlusion-induced distal fatigue.^15^^,^^19^^,^^73^ For example, Yasuda et al.^73^ were among the first to propose this after observing greater pectoralis muscle activation paired with improvements in muscle size in strength following BFR-LIX training during bench press exercise. However, in contrast to the hypothesis that increases in EMGa would result from prefatiguing of occluded distal musculature, Lambert et al.^12^ observed increased EMGa at the onset of exercise rather than after a series of repetitions. Therefore, it was hypothesized that these responses may have resulted, in part, from proprioceptive reflex mechanisms involved in detecting stretch and changes pressure (applied by the arm cuff) within muscle that may affect various chains of movement at the shoulder.^74^, ^75^, ^76^ Regarding improvements in muscle mass and strength, Bowman et al.^13^ also observed increased limb circumference and strength gains after 6 weeks of BFR-LIX training for the rotator cuff using a similar training protocol to Lambert et al.^12^ However, Brumitt et al.^77^ observed no difference between BFR-LIX and LIX alone with regards to strength or muscle thickness following twice weekly training of side-lying dumbbell external rotation. Therefore, it likely that there is a volume threshold or cumulative time under occlusion may be required to elicit proximal beneficial responses. This may require either greater exercise volumes for a given exercise or the inclusion of multiple, related exercises to elicit a desired response. Similar to the aforementioned findings with regards to upper-extremity exercise, Bowman et al.^14^ observed greater improvements in lower-extremity strength, endurance, and hypertrophy with 6 weeks of BFR-LIX compared with LIX alone. These findings were in contrast to Sakamaki et al.,^78^ who examined the proximal and distal adaptations to BFR during chronic walk training. The contrast between these 2 investigations suggests that similar to current upper-extremity findings, there may be intensity and volume thresholds required for beneficial proximal responses to occur.

### Potential Systemic Mechanisms That May Influence Proximal Responses to BFR

In addition to the potential mechanisms discussed with regard to muscle activation of tissues directly proximal to the site of occlusion, systemic anabolic signaling mechanisms (previously described) also have been hypothesized to play a role in the proximal response to BFR-LIX for muscle directly proximal to the site of occlusion, contralateral effects, and whole body cross-over effects.^13^, ^14^, ^15^^,^^21^^,^^31^ However, data on the direct measurement of these potential effects are extremely limited and, therefore, most current conclusions related to systemic action have been derived from indirect observations. For example, Bowman et al.^13^^,^^14^ observed increased grip strength measures in the contralateral limb to the BFR treatment limb following rotator cuff training and, in a separate study, observed increased thigh girth and knee extension strength in the contralateral limb to the BFR treatment limb following lower-extremity resistance exercise. Although no direct local or systemic measures of anabolic signaling were made, it was inferred that these responses may have been, in part, to systemic anabolic signaling mechanisms. Future mechanistic studies will be required to determine the degree to which systemic signaling mechanisms during and following BFR-LIX may impact tissues not subjected to occlusion. Furthermore, as systemic responses to resistance training are largely governed by training volume, work to rest intervals, metabolic cost of exercise, exercise intensity, and the amount muscle engaged in contractile activity, several factors remain unknown with regard to thresholds for the stimulus needed for a given systemic effect to determine if the use of BFR is practical for a given desired outcome. In the case of BFR-LIX, these factors included, but are not limited to, (1) bilateral versus contralateral training; (2) small muscle single joint versus large muscle multijoint exercises; (3) the total amount of exercises performed; (4) total time under occlusion; and (5) upper- versus lower-extremity exercises using considerably different quantities of contractile tissue. For example, fatiguing bilateral leg press under BFR would likely result in differential systemic responses compared to unilateral rotator cuff exercise.

**Fig 1:**
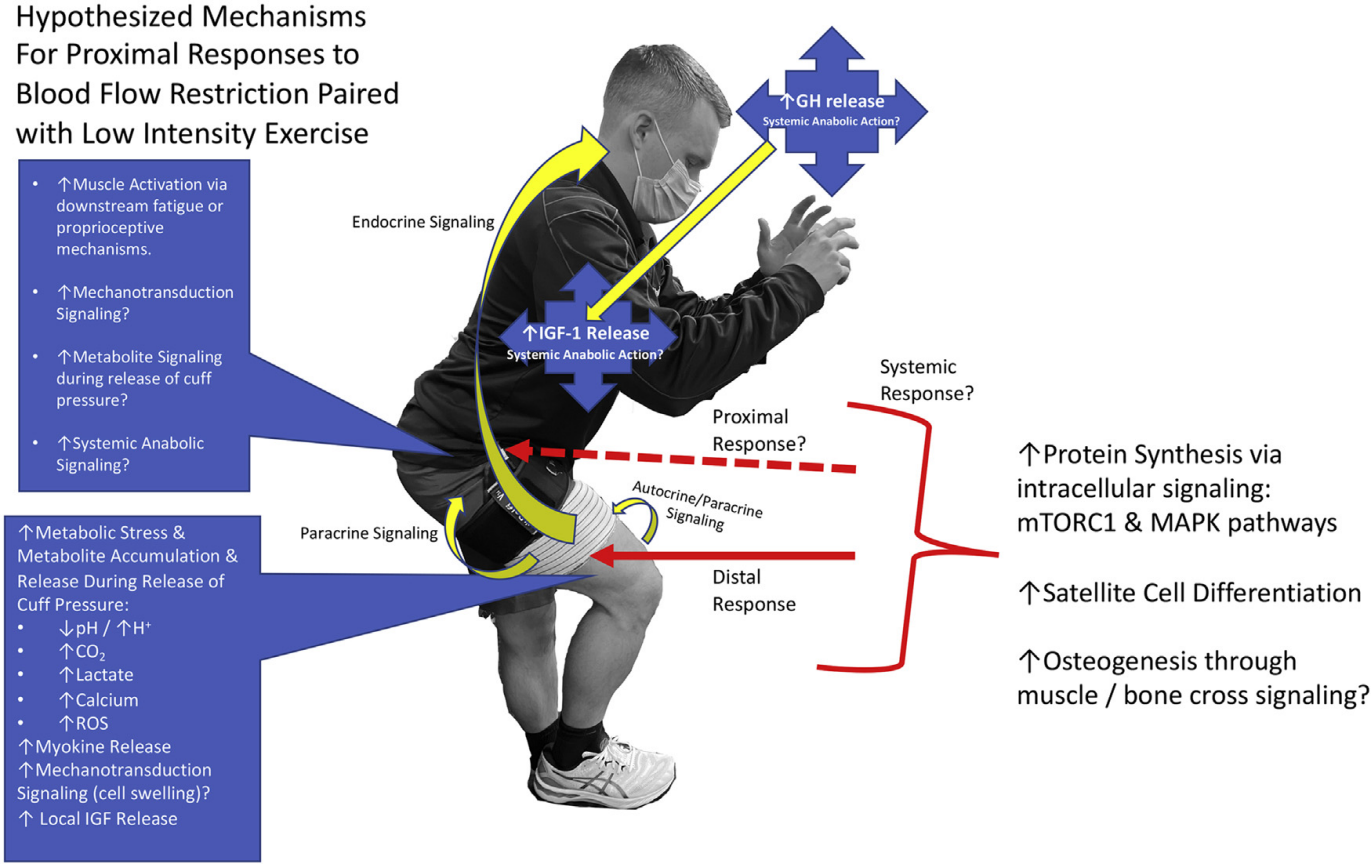
Potential mechanisms for stimulating proximal effects during blood flow restriction training paired with low-intensity resistance exercise. (GH, growth hormone; IGF, insulin-like growth factor; MAPK, mitogen-activated protein kinase; mTORC1, mammalian target of rapamycin complex 1; ROS, reactive oxygen species.)

Although several aspects of BFR remain to be investigated, the following findings provide some support (summarized in Fig 1) for its use for clinical application for treatment and preventative training for beneficial proximal outcomes.

## Current Paradigms in Clinical Use

### Initial Findings in Distal Musculature

In early publications on BFR with exercise, naturally, the largest muscles distal to the occlusive device receive a majority of the attention. The quadriceps is one particular muscle group popular for research due to its size and dominance in functional lower extremity activities.^5^^,^^6^^,^^10^^,^^11^ An early study by Eiken et al.^79^ examined the effect of “ischemic training” on quadriceps femoris muscle activity during a single-leg cycling trial. They found that blood flow-restricted training induces “…an increase in the share of the muscle cross-sectional area…” indicating the potential for ischemically induced changes in the metabolic characteristics of the muscle.^79^ These findings were likely instrumental in setting the stage for further study into quadriceps function with BFR nearly two decades later. Cook et al.^2^ found that lower loads could be used with knee extensions and BFR to elicit a similar fatigue effect in the quadriceps versus high-load exercise. The potential for significant muscle strength gains (or at least atrophy mitigation) incites significant attention from physical therapists as rehabilitation protocols are typically designed around these facets. More recent work, including a systematic review with meta-analysis from Van Cant et al.,^80^ build on the premises from authors previous, indicating that “BFR could be a useful option for patients with knee conditions where conventional quadriceps strengthening program exacerbate knee symptoms.” Undoubtedly, the findings from these studies are promising to rehabilitation professionals, especially when considering quadriceps activity and knee function. However, a common cautionary plea in the early literature indicates a need for further research into pathologic individuals, especially those involved in postsurgical cases.

### Postsurgical Potential

Postoperatively, patients encounter significant muscle loss due to disuse atrophy, traumatic inhibition, and edema.^6^^,^^11^^,^^81^ This “muscle dump” coincides with significant strength and function impairments that often take months to years to regain.^6^ BFR has taken a recent precedent in the literature involving postsurgical cases and the subsequent recovery and rehabilitation. ACL reconstruction involves prominent quadriceps weakness following surgery, greatly limiting patients in the acute stages of rehab.^5^ Furthermore, suboptimal function of the knee extensors can lead to an unsuccessful return to sport, reinjury, or the development of knee arthritis later in life.^82^, ^83^, ^84^ To mitigate the postsurgical atrophy seen in patients with ACL injury, physical therapists recently have seen significant improvements in the early phases of rehabilitation with the use of BFR.^11^^,^^85^ Typically, patients are performing low-level exercises early in the rehabilitation process (such as quadriceps or hamstrings isometrics) in an effort to regain neuromuscular control and activation.^5^ Furthermore, some surgical protocols (especially those involving cartilaginous interventions) require significant periods of partial to complete reduction of weight-bearing through the limb.^25^^,^^86^ Several studies have observed positive benefits in exercises involving limited weight-bearing scenarios, which hold promise to be advantageous for patient progress and function.^25^^,^^86^, ^87^, ^88^ Similar to ACL reconstruction and other lower-extremity orthopaedic surgery, total joint replacement in the lower extremity lends to significant functional decline and impairment.^89^, ^90^, ^91^, ^92^, ^93^ Total knee and total hip arthroplasty are becoming more popular for the aging population. Individuals frequently suffer up to an 80% loss in knee-extension strength in the initial days to weeks following surgery.^89^, ^90^, ^91^ Generally, the trauma from the surgery is responsible for the pain and diminished function, but most patients can perform light exercise and bear weight through the limb within 24 hours postoperatively.^92^^,^^93^ Currently, randomized controlled trials with BFR for individuals following an arthroplasty are limited and require further research. The lower-intensity nature of BFR and exercise may be enticing for this population in the future.

### Aerobic Considerations

While this review has discussed important considerations for muscular strength, we must also consider recent introspect into the antithetical realm—aerobic capacity. Greater degrees of aerobic fitness have been shown to improve sports performance, increase rehabilitation potential, and improve general health.^94^, ^95^, ^96^ Similarly to strength training, improving aerobic performance requires significant training at a high threshold. Often, individuals must incorporate activities which place them at or above 70% of their maximal oxygen consumption (VO_2_max) to achieve a successful training effect.^97^^,^^98^ Unfortunately, many circumstances, such as poor health, injury, surgery, lack of time, and/or resources, can hinder one’s ability to incorporate aerobic activities into their regimen.^99^^,^^100^ Interestingly, BFR seems to have found its way into the aerobic environment, establishing similar positive effects seen in anaerobic training. Bennett and Slattery^101^ performed a systematic review on the effects of BFR on aerobic capacity and performing exercises such as continuous cycling, treadmill walking, and interval training. Interestingly, they highlighted that 8 of the 11 studies indicated positive improvements in VO_2_max or VO_2_peak.^101^ The studies that did not find significant benefits included older subjects or used much lower occlusive pressures.^101^ The modality of exercise does not seem to influence the observed changes in aerobic capacity.

### Pain Modulation

Pain is a significant limiting factor for patients of all backgrounds and pathologies when in rehabilitation. Physical therapy interventions, inclusive of exercise, are often directly targeted at pain signaling due to acute injury, chronic musculoskeletal disorders, and postsurgical effects. Moderate-intensity exercise has been shown to actually induce an analgesic effect.^8^, ^9^, ^10^^,^^102^ However, if an individual suffers from pain during exercise, this can limit their productivity, reducing the potential to achieve exercise-induced hypoalgesia.^8^, ^9^, ^10^ Otherwise, therapeutic activities often are structured in an effort to avoid pain or increased discomfort. Korakakis et al.^10^ found clinically significant reductions in anterior knee pain for a sample of patients after low-level exercise with BFR. They found that the reduction in pain was relatively long-lasting as well (>45 minutes), which allowed for greater compliance and consistency with physical therapy activities.^10^ Various mechanisms behind this analgesic effect have been explored, including opioid and endocannabinoid-mediated pain inhibition, conditioned pain modulation, recruitment of high threshold motor units, exercise-induced metabolite production, and an interaction between cardiovascular and pain regulatory systems.^8^ Song et al.^102^ performed a systematic review of similar studies that examined these theoretical mechanisms and concluded that exercise with BFR may serve as an effective method of pain management for those who cannot train with higher loads. However, “...the roles of these mechanisms are still unclear and require further clarification.”

### Neurologic/Systemic Disorders

Orthopaedic pathology and procedures naturally hold a strong spotlight in the literature and clinical use of BFR with exercise. We often think of rehabilitation of muscle, bone, and connective tissue disorders as a relatively straightforward continuum with predictable outcomes and time frames. However, muscle weakness, pain, and dysfunction are also hallmarks of certain neurologic or autoimmune diseases as well. For these individuals, similar rehabilitation principles hold true—build strength and mitigate atrophy/weakness as much as possible. Some notable studies have already set the groundwork for the use of BFR in patient populations outside of the orthopedic and sports realms. Liang et al.^103^ and Jørgensen et al.^104^ have performed similar randomized control trials in patient populations with sporadic inclusion-body myositis. Both groups examined similar variables in their patients—quality of life, strength, self-reported function, and endurance. Liang et al.^103^ found favorable results for the BFR group in all variables. Jørgensen et al.^104^ did not note significant differences in self-reported or objective outcomes, but they did find that the BFR group did not encounter diminished strength versus the controls. Thus, concluding that BFR with exercise may have a “preventive (retaining) effect on the disease-related decline in leg muscle strength, which may aid the long-term preservation of physical function and postpone the need for healthcare assistance.”^104^ Furthermore, Mattar et al.^105^ found favorable results in quality of life, muscle strength, and function by using BFR and exercise with patients diagnosed with polymyositis and dermatomyositis. Muscle mass was also improved in their BFR group, which may indicate added benefit for these groups.^105^ Finally, Douris et al.^106^ studied BFR with exercise in an individual with Parkinson disease. Their outcomes were positive for improvements in strength and function as well as Parkinson-related outcome measures for quality of life.^106^ While a litany of research is becoming available in the orthopedic literature, the future of BFR may expand into a number of other populations as well.

## The Future of BFR: Proximal Potential

Previous mechanistic models for BFR with exercise have favored distal structures and muscle groups over proximal regions such as the shoulder and hip. As further research is conducted, we are gaining a better perspective on how BFR may benefit a wider variety of clinical cases and the population.

The shoulder is a complex structure which requires a delicate balance between large and small muscles to establish a force couple of the scapulothoracic and glenohumeral architecture.^107^ This provides a proximally stable foundation for a distally mobile limb to perform unencumbered tasks such as reaching, pushing, pulling, or throwing.^107^^,^^108^ The upper extremity is typically active in both open- and closed-chain scenarios, requiring notable strength and proprioception to avoid injury. In physical therapy and rehabilitation, careful consideration is warranted to restore function to the shoulder region and usually consists of meticulous strengthening exercises using progressive loading.^107^^,^^108^ The inclusion of BFR with lower loads for the shoulder and associated structures is of particular interest in an effort to potentially accelerate rehabilitation, minimize adverse effects of postsurgical conditions, and to potentially prevent injury to a vulnerable region. Unfortunately, few studies exist examining the effect of BFR on the proximal muscles including the rotator cuff and periscapular musculature. As mentioned previously, Yasuda et al.^73^ first elucidated the positive effects of BFR for the pectoralis major following 8 weeks of bench press training. Lambert et al.^12^ performed a randomized-controlled trial with multiple low-intensity exercises for the rotator cuff, observing strength, endurance, muscle activity, and lean-mass changes between groups either using BFR or not. Dankel et al.^15^ found similar results for proximal and distal muscles in the upper extremity, noting that the inability to directly occlude certain muscles does not limit their potential to achieve strength gains at lower loads.

Similar to the shoulder, the hip remains a vital region to the active population. A prominent weight-bearing structure, the femoroacetabular joint and adjoining musculature are highly active with many functional movements and sports-related activities.^17^ The gluteus maximus, medius, and minimus each provide a unique role in establishing a powerful, yet stable synergy for high performance mobility.^17^ Furthermore, the hip serves as a crucial component to the lower-extremity kinetic chain as deficits in hip strength, power, or endurance are predisposing factors for knee, ankle, or foot injuries.^17^ The role of BFR in lower-extremity rehabilitation has been well-documented, but primarily for strength and function of muscles distal to the occlusion stimulus. The hip seems to hold significant potential in this realm as weight-bearing is often limited following severe injury or surgery and the resultant muscle atrophy and weakness is profound.^90^ However, direct study for the hip musculature is severely understudied at this time. Bowman et al.^14^ again examined proximal, distal, and contralateral limb strength and circumference with randomized groups exercising with (or without) BFR. Similar to their upper-extremity results, the data favor BFR with lower-extremity exercise, even proximally at the hip.^14^ As of this review, no other published data exist for the gluteal or intrinsic hip musculature with BFR and exercise.

## Clinical Utility and Practice: From Bench to Bedside

Conceptual and mechanistic models for the clinical use of BFR have been widely discussed and studied over the past decade. Many articles have a focal intent in their research (strengthening versus aerobic fitness vs hormonal/systemic responses) but provide a multitude of methods or techniques with no one globally-accepted protocol available. However, recent systematic reviews and meta-analyses are progressively merging similar findings to provide clinicians with greater consistency in clinical application.^1^^,^^4^^,^^15^^,^^22^^,^^101^^,^^109^ Table 1 indicates current clinical recommendations for strength training with BFR based on this review. Currently unknown are how differentiation in these protocols affect proximal versus distal musculature to a greater degree. Table 2 includes selected activities prevalent in the literature and clinical practice targeted at vital proximal musculature that take precedence in upper- or lower-extremity rehabilitation. To date, no studies have examined EMG activity of the lower extremity proximal musculature during the proposed exercises. Lambert et al.^12^ are the first to elucidate EMG signaling of the proximal musculature of the upper extremity during BFR with exercise targeted at the rotator cuff over an 8-week interventional design.

**Table 1:** Recommended Strengthening Parameters

Clinical Recommendations for Strengthening^1^^,^^4^^,^^15^^,^^22^		
	Upper Extremity	Lower Extremity
Occlusive pressure	50% LOP	80% LOP
Resistance/intensity	30%-50% 1RM, MVC	20%-30% 1RM, MVC (7-8 RPE)
Repetitions/sets	30/15/15/15, 30 second rest-periods	30/15/15/15, 30 second rest-periods
Frequency	2-3×/wk	2-3×/wk
Duration	8+ wk	8-12 wk

1RM, 1-repetition maximum; LOP, limb occlusion pressure; MVC, maximal voluntary contraction; RPE, rating of perceived exertion.

**Table 2:** Exercise Selection for Strengthening

Therapeutic Exercise Selection for Strengthening^1^^,^^11^, ^12^, ^13^, ^14^			
Upper Extremity		Lower Extremity	
Muscle Group	Exercise	Muscle Group	Exercise
Rotator Cuff	• Internal rotation at 0° of abduction • External rotation at 0/90° of abduction • Side-lying external rotation • Prone internal/external rotation at 90° • Standing flexion and scaption	Gluteus maximus	• Prone straight leg raise • Quadruped hip extension • Bridges, single leg bridges • Squats, single leg squats • Leg press • Lunges • Forward step ups
Periscapular musculature	• Resisted rows • Shoulder extension • I’s/T’s/Y’s • Serratus walk-ups • Resisted shoulder retraction with external rotation	Gluteus medius/minimus	• Sidelying straight leg raise • Clamshells • Quadruped fire hydrants • Lateral band walks • Standing hip abduction • Lateral step ups
Chest/pectorals	• Barbell/dumbbell press • Push ups • Dumbbell fly	External/internal rotators	• Seated external/internal rotation • Prone external/internal rotation

In addition to strengthening, aerobic activities remain commonplace in rehabilitation and physical therapy dependent on patient goals and needs. Various trials have been performed with BFR and aerobic exercise, generally indicating that this combination produces improvements in aerobic performance and fitness in various populations irrespective of training intensity.^101^^,^^109^ Interestingly, there may be differences in observed adaptations based on age, however.^101^ Table 3 outlines current clinical recommendations for aerobic activities with BFR. Note that BFR with upper-extremity aerobic activities (arm ergometer, swimming) is vastly understudied.

**Table 3:** Recommended Aerobic Parameters

Clinical Recommendations for Aerobic Activities^101^^,^^109^	
Occlusive pressure	50%-80% LOP
Resistance/intensity	30%-40% VO_2_max
Time	10-30 min
Frequency	3-4×/week
Duration	6-8+ weeks

LOP, limb occlusion pressure; VO_2_max, maximal oxygen consumption.

Finally, a relatively newer method of using BFR for the benefit of patient care is known as ischemic preconditioning (IPC). The concept of IPC has been observed throughout the medical literature for a number of years and can be defined as the exposure to brief periods of circulatory occlusion and reperfusion to protect local or systemic organs against subsequent bouts of ischemia.^110^ Furthermore, we have seen studies find an improvement in VO_2_max, increased sports performance, and increased strength/endurance.^110^, ^111^, ^112^, ^113^, ^114^, ^115^ Franz et al.^116^ also found that IPC may blunt exercise-induced muscle damage when performed before bouts of eccentric exercise of the muscle flexors. Tanaka et al.^117^ theorize that the origin of the beneficial effects from IPC may likely be the enhancement of mitochondrial metabolism in skeletal muscle. Naturally, the occlusive stimulus used with BFR makes sense when considering the potential systemic and local effects of IPC. Typically, most protocols involve full occlusion without the addition of exercise. Table 4 provides a current guideline for IPC of either the upper or lower extremities.

**Table 4:** Recommended Ischemic Preconditioning (IPC) Parameters

Clinical Recommendations for IPC^110^	
Occlusive pressure	100%
Exercise	None, static
Time/duration	5 min of occlusion 5 min break
Sets	3-4
Frequency	3-5×/wk
Duration	6-8 wk

## Injury Prevention and Recovery

Sports participation in the United States is constantly growing and evolving. Professional sports account for a multibillion-dollar industry and consist of some of the highest skilled athletes in the world. With some athletes now signing contracts for several hundred million dollars (USD), the science that goes into their performance is extremely complex and vital for teams to see a return on their investment. Immense research has been performed on performance optimization and many studies will allude to a delicate balance of training and recovery. The ability to reduce physical and mental stress while remaining productive in training regimens plays a key role in an athlete or teams’ success. The potential for BFR to meet these needs might be the “holy grail” for future performance research.

The sport of basketball is an international sensation, widely popular with tall and athletic individuals in an extremely competitive environment. A vast majority of injuries encountered in the sport are about the lower extremity and can be directly correlated to fatigue and intensity. Several researchers have studied the effect of competitive basketball games on fatigue levels and most indicate a cumulative workload effect, especially with multiple games played per week.^118^, ^119^, ^120^, ^121^ Furthermore, Rubin et al.^118^ detected significant seasonal changes in the articular cartilage of basketball players and regional differences in the articular cartilage that are indicative of basketball-specific stress on the femoral cartilage. Thus, maintaining adequate muscle mass and strength about the lower extremities can be particularly challenging for the basketball player, especially in a full 82-game season as played in the National Basketball Association. Players experience even more demand in playoff situations with congested schedules (games every other day) when in need of their absolute peak performance.^121^ Using BFR with lower loads and mitigating loss of Type II muscle would be highly beneficial for basketball athletes. Additionally, Faltus et al.^122^ provide further theoretical concepts for BFR within the basketball population, inclusive of chronic pain management, increased motor unit recruitment, and kinetic chain/proximal improvements. Further study is needed within these athletes to determine appropriate loading parameters as well as exercise selection and frequency.

Baseball is another sport with extremely long and stressful competitive seasons. Major League Baseball players are subjected to 162 games in a regular season with multiple series in playoff rounds. These athletes encounter significant stress/strain over the course of competition, producing some of the highest angular velocities recorded at the shoulder and elbow while throwing at maximum intensity. Khalil et al.^123^ and Chalmers et al.^124^ discovered anatomical changes about the shoulder and elbow that occur in the baseball athlete during a long season. More specifically, the ulnar collateral ligament exhibits a thickening effect, potentially in relation to a protective response to the large amounts of valgus stress in the elbow during throwing.^124^ Furthermore, significant decreases in range of motion are common, especially in the shoulder, towards the end of the season.^124^^,^^125^ Therefore, to protect from injury or time lost, baseball athletes need to maintain optimal strength without adversely affecting performance or tissue quality. BFR has been shown to improve strength, endurance, and lean mass about the upper extremity with relatively low loads which would be particularly helpful for the baseball athlete.^12^ With future study toward optimal training loads to performance enhancement, we can better understand how the role of BFR may expand in the sport of baseball, especially for proximal musculature such as the shoulder.

Ultimately, professional and amateur sports can place large demands on the human body. Athletic trainers, physical therapists, and performance specialists are tasked with the role of helping athletes maintain peak performance while mitigating injury risk. The inclusion of BFR regimens seems to hold promise, especially for proximal musculature due to the inherent low loads utilized during training.

## Conclusions

In conclusion, the combination of BFR-LIX seems to promote an improvement in skeletal muscle anabolism at low loads to mitigate atrophy postinjury or surgery and improve strength while minimizing risk. Interestingly, benefits have been found in musculature proximal to the occlusive stimulus that may lend promise to a greater variety of patient populations and conditions. Furthermore, an increasing demand for BFR use in the sports world warrants further research for performance research and recovery.

## Supplementary Data

ICMJE author disclosure forms
